# Rehabilitation Training Can Significantly Increase the Serum IL-11 Levels and Improve the Prognosis in Ischemic Stroke Patients

**DOI:** 10.1155/2023/1865760

**Published:** 2023-02-22

**Authors:** Xiaoliu Li, Jing Zhang, Qiang Wang, Lian Xiang, Jing Dong

**Affiliations:** ^1^Department of Rehabilitation Medicine, Minhang Hospital Affiliated to Fudan University, Shanghai 201199, China; ^2^Department of Rehabilitation Medicine, The First Affiliated Hospital of Hainan Medical University, Haikou 201199, China; ^3^Department of Cardiothoracic Surgery, Zhoupu Hospital Affiliated to Shanghai Medical College of Health, Shanghai 201318, China; ^4^School of Electronic and Information Engineering, Soochow University, Suzhou, China; ^5^Center of Diagnosis and Treatment for Cervical Diseases, Obstetrics and Gynecology Hospital of Fudan University, Shanghai 200011, China

## Abstract

We aimed to explore the expression of IL-11 in ischemic stroke patients and its correlation with rehabilitation training and prognosis. The present randomized control study recruited ischemic stroke patients who were admitted during March 2014 to November 2020. All patients underwent computer tomography (CT) and magnetic resonance imaging (MRI) examination. All patients were randomly divided into two groups, including rehabilitation training (RT) group and control group. The patients in the RT group were received rehabilitation training within 2 days after the vital signs were stable while control group received routine nursing. The serum interleukin- (IL-) 11 levels were measured by enzyme-linked immunosorbent assay (ELISA) when patients were just hospitalized and 6 h, 24 h, 48 h, 72 h, and 90 h after treatment. Demographic, clinical statistics, imaging data, and the National Institutes of Health Stroke Scores (NIHSS) were recorded. The modified Rankin Scale (mRS) scores were measured after 90 days treatment to assess the prognosis of ischemic patients. The serum IL-11 levels of the RT group elevated more quickly during the study time compared with the control group. In addition, the NIHSS and mRS scores of ischemic stroke patients in the RT group were significantly lower than that in the control group. The NIHSS score, the proportion receiving rehabilitation training, and the levels of IL-11, triglyceride (TG), and high-density leptin cholesterol (HDLC) of ischemic stroke patients in the mRS score ≥ 3 group were remarkably elevated than that in the mRS score ≤ 2 group. However, the serum IL-11 levels of ischemic stroke patients were obviously decreased in the mRS score ≥ 3 group. IL-11 could be a potential diagnostic biomarker of poor prognosis of ischemic stroke patients. Furthermore, IL-11, NIHSS score, and rehabilitation training were the risk factors for poor prognosis of ischemic stroke patients. This study demonstrated that the ischemic stroke patients in the RT group had higher serum IL-11 levels and better prognosis. This study might provide a new approach to improve the prognosis of patients with ischemic stroke. This trial is registered with ChiCTR-PNR-16007706.

## 1. Introduction

As a major reason of permanent disability [1, 2], stroke influences 13.7 million people worldwide and causes 5.5 million deaths each year [3, 4]. Stroke is the leading cause of disability in adults, and about 90% stroke patients leave some residual defects [5]. Ischemic stroke patients account for about 85% of all stroke patients [6]. Many risk factors are associated with ischemic stroke, including smoking [7], cardiac causes [8], hypertension [9], diabetes mellitus [10], oxidative stress [11], and inflammation [12, 13]. In view of the high disability rate of ischemic stroke, improving the prognosis of patients is important for the treatment of ischemic stroke.

Posttreatment rehabilitation is the most common method to improve the prognosis of ischemic stroke patients [14, 15]. In addition, inflammation after ischemia-reperfusion injury is considered as an inevitable pathological process in postischemic brain injury [16]. Dynamic changes in the release of several proinflammatory and anti-inflammatory cytokines in the brain may influence the progression of ischemic stroke. Therefore, these inflammatory factors are considered as biomarkers of the pathogenesis and prognosis of stroke [17]. However, the importance of these biomarkers in predicting the prognosis of ischemic stroke patients still needs further investigation. Interleukin-11 (IL-11) is a soluble factor in the supernatant of plasma cell tumor-stimulating cells [18]. IL-11 has many functions *in vivo*, including anti-inflammatory, cardioprotective, and reducing apoptosis [19]. It has been reported that IL-11 plays a protective role in ischemia-reperfusion injury of the heart, kidney, and intestine [20, 21]. A recent mouse model restriction of cerebral ischemia-reperfusion injury found that IL-11 was declined in the cerebral ischemia model, and the upregulation of the IL-11 expression could improve the cerebral ischemia neuropathy injury and neurological function score [22]. However, there is no clinical study on IL-11 in ischemic stroke patients.

In this randomized control research, we aimed to explore the expression of IL-11 in ischemic stroke patients and its correlation with rehabilitation training and prognosis. This study might reveal the clinical significance of IL-11 in ischemic stroke patients, as well as provide novel research targets for ischemic stroke treatment.

## 2. Methods

### 2.1. Subjects

The present randomized control study recruited 404 ischemic stroke patients who were admitted during March 2014 to November 2019, and all patients underwent computer tomography (CT) and magnetic resonance imaging (MRI) examination. The criteria for inclusion were as follows: (1) the ischemic stroke was diagnosed by CT or MRI according to the Chinese guidelines for diagnosis and treatment of acute ischemic stroke 2018 [23]; (2) age ≥ 18, first onset, and admission within 48 h; and (3) patients could complete the scale assessment. The exclusion criteria included the following: (1) hemorrhagic stroke; (2) patients who were unable to complete the examination and follow-up; (3) patients with seriously infection, severe liver, renal, malignancy, and cardiovascular dysfunctions; (4) patients with epilepsy, Parkinson's disease, or other neuropsychiatric disorders; and (5) patients with important organ failure, such as liver and kidney. Written informed consent was obtained from all participants. This research had obtained approval from the ethic committee of the Zhoupu Hospital Affiliated to Shanghai Medical College of Health and kept compliance with the Declaration of Helsinki.

All patients were randomly divided into two groups using a computer-generated list by Rv, including rehabilitation training (RT) group and control group. Uniform formula uses SPSS software (SPSS Inc., Chicago, USA). For calculation of sample size, the formula of [(*tα* + *tβ*)*s*]2/*δ* was used. We used the NIHSS scores 3 months after admission as the main variable and the difference of NIHSS scores between two groups at least 1 as effective. The mean NIHSS scores were about 5 ± 3 after 3 months after admission according to the clinical experience. Thus, *δ* = 1, *s* = 3, *α* = 0.05, and *β* = 0.10. And the minimal sample size was 189.

### 2.2. Treatment and Rehabilitation Training

All patients were treated with thrombolytic therapy, antiplatelet aggregation, improving circulation, neuroprotection, scavenging free radicals, and statins according to the Chinese guidelines for diagnosis and treatment of acute ischemic stroke 2018 [23]. The patients in the RT group were received rehabilitation training within 2 days after the vital signs were stable. Rehabilitation training was as follows: sitting and lying position training; improving joint range of motion, muscle strength enhancement training, and gait training; improving the range of motion of the joint by maintaining good limb position, turning over, transfer training, and passive limb movement; cognitive ability training; and social adaptability training. Refining the selection and intensity of rehabilitation training items based on individual patients' conditions, and implementing a continuous 14-day training program, the control group received routine nursing in hospital. If the RT group patients given up training midway or the patients in the control group spontaneously carried out rehabilitation training, they would be excluded from this study. All patients were followed up for 3 months.

### 2.3. Blood Sampling Measurement

The serum IL-11 and other inflammatory factors levels were measured by enzyme-linked immunosorbent assay (ELISA). Blood samples of fasting cubital venous (5 mL) were collected within 24 h after admission for all cases. Samples were centrifuged at 2000 g for 15 min, following with ELISA tested using commercially available kits (IL-11 EK0419 BOSTER, sensitivity < 10 pg/mL, detection range 31.2 pg/mL-2000 pg/mL, IL-6 MBS175877 MyBioSource, CRP MBS177184 MyBioSource, TNF-*α* MBS824943 MyBioSource). The serum cytokine levels were measured when patients were just hospitalized and 24 h, 48 h, 72 h, and 90 h after treatment.

### 2.4. Data Collection and Scale Scoring

Demographic and clinical statistics including age, BMI, sex, smoke condition, diastolic blood pressure (DBP), systolic blood pressure (SBP) and history of hypertension, hyperlipidemia, and diabetes were collected. Using an automatic biochemical analyzer to performed whole blood test by Hitachi 7600 of Hitachi Corporation, the total cholesterol (TC), fasting plasma glucose (FPG), triglyceride (TG), low-density leptin cholesterol (LDLC), and high-density leptin cholesterol (HDLC) levels were recorded. Imaging indexes of MRI and CT including infarct location, infarct volume, multiple or single, and vulnerable plaque were collected. The National Institutes of Health Stroke Scores (NIHSS) were recorded when the subjects were hospitalized to assess the severity of stroke. Using the modified Rankin Scale (mRS) to assess the prognosis after 90 days of treatment, mRS score ≥ 3 indicated a bad prognosis, and mRS score ≤ 2 indicated a good prognosis.

### 2.5. Statistical Analysis

Data were expressed by mean ± SD or median (range) according to distribution, which was confirmed by Kolmogorov-Smirnov analysis. Mann–Whitney test or Student's *t* test was used for comparison between two groups. Chi-square test was used for rates. ROC curves assess the diagnostic value of IL-11 for poor prognosis of ischemic stroke patients. Logistic regression was performed for risk factors of bad outcome of ischemic stroke. *P* < 0.05 is regarded as significantly different. All data used SPSS 18.0 to statistical analyses.

## 3. Results

### 3.1. Clinical Characteristics of All Participants

This study enrolled 404 ischemic stroke patients. All patients were randomly divided into the RT group (*n* = 197) and control group (*n* = 207). The basic characteristics of two group patients were shown in Table 1. About 70% of ischemic stroke patients had hypertension, 30% had hyperlipidemia, and about 45% had hyperglycemia. There was no significant difference between the two groups in terms of basic clinical information and expression of serum cytokine markers at the time of admission.

### 3.2. Serum IL-11 Expression and Clinical Data in Each Group during the Study Time

**Figure 1:**
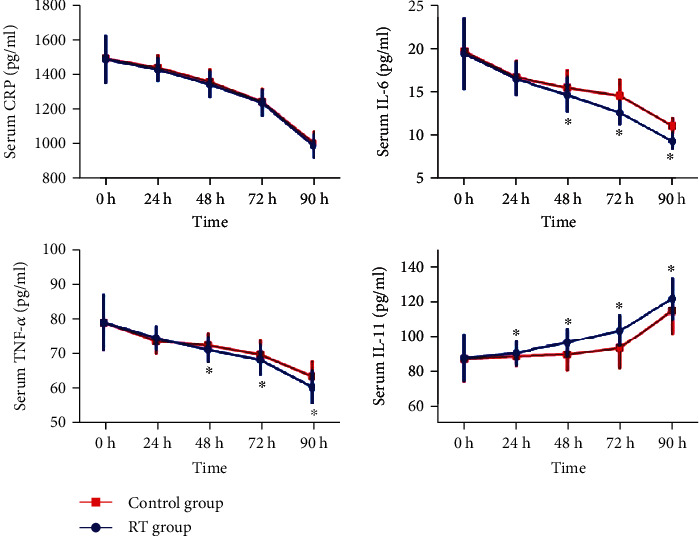
Comparisons of serum IL-11 and other inflammatory factors levels between two groups. Data were expressed by mean ± SD. The continuous data were compared using Student's *t* test between two groups. All data were normally distributed.

**Figure 2:**
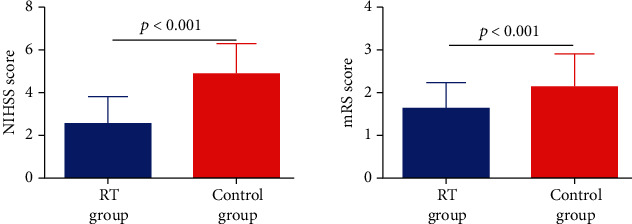
Prognosis of ischemic stroke patients in RT and control group. Data were expressed by median (range) and analyzed by Mann–Whitney *U* test. All data were nonnormally distributed.

Then, we draw line graphs of all subjects to show the dynamic variations of the IL-11 expression. It was observed that the IL-11 levels were increased gradually with treatment in both groups (Figure 1). We found no obvious differences of the IL-11 expression between two groups when the patients were just hospitalized. However, the serum IL-11 levels of the RT group elevated more quickly during the study time (*P* < 0.05), while IL-6 and TNF-*α* decreased quickly in the RT group. To further investigate the correlation between the recovery of ischemic stroke patients and rehabilitation training, we measured NIHSS and mRS scores after 90 days treatment. The results indicated that the NIHSS and mRS scores of ischemic stroke patients in the RT group were significantly lower than that in the control group (Figure 2, *P* < 0.05). Spearman's analysis showed that IL-11 was negatively correlated with NIHSS and mRS scores (Table 2).

### 3.3. Connection of Serum IL-11 Levels, Clinical Data, and Prognosis of Ischemic Stroke Patients

All patients measured mRS scores after 90 days of treatment and divided into two groups including the mRS score ≤ 2 group and mRS score ≥ 3 group. Compared with the demographic and clinical data of two groups when the patients were hospitalized, we found no significant differences in age, sex, BMI, smoking proportion and levels of FPG, TC, and LDLC between the mRS score ≤ 2 group and mRS score ≥ 3 group (Table 3). The NIHSS score, infarct volume, and the levels of IL-6, TG, and HDLC of ischemic stroke patients in the mRS score ≥ 3 group were remarkably elevated than the ischemic stroke patients in the mRS score ≤ 2 group (*P* < 0.05). In addition, the serum IL-11 levels of ischemic stroke patients were obviously decreased in the mRS score ≥ 3 group.

### 3.4. Diagnostic Value of IL-11 for Poor Prognosis of Ischemic Stroke Patients

**Figure 3:**
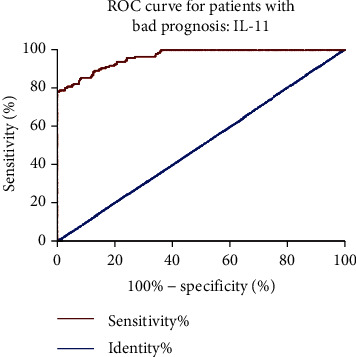
ROC curves for IL-11 in diagnostic poor prognosis of ischemic stroke patients.

We draw ROC curves to assess the diagnostic value of IL-11 for poor prognosis of ischemic stroke patients. The result showed that IL-11 could be a potential diagnostic biomarker of poor prognosis of ischemic stroke patients (Figure 3), the AUC of IL-11 was 0.965, cutoff value was 87.31 pg/mL, sensitivity was 87.5%, and specificity was 87.2%.

### 3.5. Risk Factors of Poor Prognosis of Ischemic Stroke Patients by Logistic Regression Analysis

Finally, the risk variables for poor prognosis in ischemic stroke patients were calculated using binary regression analysis. It was found that IL-11 (95% CI 0.346~0.799, *P* = 0.03), NIHSS score, infarct volume, TG, HDLC, and IL-6 were the risk factors for bad prognosis of ischemic stroke patients (Table 4).

## 4. Discussion

Although intravenous thrombolysis and thrombectomy can be effective in the treatment of ischemic stroke, progressive neuronal degeneration and functional loss remain difficult to resolve during treatment and rehabilitation. Cerebral ischemia promotes the production of proinflammatory mediators and induces cell death and cell dysfunction, thus inducing neuroinflammation. Excessive inflammation can produce neurotoxins and brain edema. Recently, poststroke immune response has recently a new breakthrough goal in the treatment strategy of ischemic stroke [24, 25]. In our present research, we found that the serum levels of IL-11 were declined in ischemic stroke patients with bad prognosis.

Rehabilitation training can improve the prognosis and daily living of ischemic stroke patients. A nationwide retrospective cohort study in Japan confirmed that early rehabilitation training can improve the daily living of ischemic stroke patients [26]. The relationship between rehabilitation training and changes of inflammatory factors has been controversial. Previous study found that the rehabilitation after ischemic stroke depended on the start time of exercise, and premature exercise after stroke leads to increased expression of proinflammatory factors [27]. Another research showed that rehabilitation training could not reduce the expression of inflammation [28]. In our research, the results indicated that the serum IL-11 levels of the RT group elevated more quickly during the study time.

Some biomarkers have been found correlated to the prognosis of ischemic stroke patients. Kwan et al. found that the increasing of IL-6 was related to the severity and infection of ischemic stroke patients, and serum IL-6 levels could predict the mortality in the first two years after stroke [29]. Wang et al. supported that high macrophage migration inhibitory factor levels were independently related to the severity of ischemic stroke patients, as well as the bad prognosis [30]. Li's study showed that the serum IL-4, IL-5, IL-7, and IL-9 levels decreased in the ischemic stroke patients with poor outcome [30]. Wang et al. confirmed that the plasma high mobility group box protein 1 has a satisfactory predictive value for cerebral ischemia-reperfusion injury in ischemic stroke patients [31]. IL-11 is a multifunctional cytokine which is involved in the development of various diseases [21, 32]. Yang and Shao's study showed that the serum IL-11 levels were correlated with the severity of hypertensive intracerebral hemorrhage patients [33]. Ren et al. found that IL-11 was a biomarker in diagnosis of pancreatic cancer, and it might be used to predict the prognosis of pancreatic cancer patients [34]. Zhang et al. [22] and Obana et al.'s [35] animal studies confirmed that IL-11 played a protective role in ischemia-reperfusion injury. In the present study, we also demonstrated for the first time in a clinical trial that IL-11 was progressively elevated in recovering of ischemic stroke patients and correlated with cytokines and prognosis of ischemic stroke patients.

### 4.1. Limitations

This present research also has some limitations. First, this is a single-center study. Secondly, we only checked a small number of biomarkers. Thirdly, the molecular mechanism of IL-11 affecting ischemic stroke development is unclear.

## 5. Conclusion

In summary, the present study showed that the ischemic stroke patients in the RT group had higher serum IL-11 levels and better prognosis, indicating that rehabilitation training might improve the prognosis of ischemic stroke patients and elevate the levels of IL-11. This study may provide a new approach to screen ischemic stroke patients with worse prognosis in advance, as well as provide a new approach to improve the prognosis of ischemic stroke patients.

## Figures and Tables

**Table 1:** Basic characteristics of all patients.

Variable	RT group (*n* = 197)	Control group (*n* = 207)	*P*
Age, y	59 (44~74)	58 (41~79)	0.289
Sex, female (%)	95 (48.22)	101 (48.97)	0.999
BMI	25.58 ± 2.18	25.65 ± 2.17	0.771
Current smoker, *n* (%)	76 (38.58)	85 (41.06)	0.885
NIHSS	7 (1~22)	6 (1~20)	0.903
Hypertension, *n* (%)	138 (70.05)	149 (71.98)	0.876
Hyperlipidemia, *n* (%)	34 (17.26)	32 (15.46)	0.847
Diabetes, *n* (%)	41 (20.81)	48 (23.19)	0.865
Infarct volume (cm^3^)	1.02 (0.20~4.98)	1.04 (0.19~5.05)	0.938
Infarct location			
Left, *n* (%)	101 (51.27)	121 (58.45)	0.394
Right, *n* (%)	71 (36.04)	67 (32.38)	0.654
Both sides, *n* (%)	25 (12.69)	19 (9.18)	0.499
FPG (mmol/L)	6.81 ± 0.59	6.85 ± 058	0.479
TC (mmol/L)	4.33 ± 0.74	4.31 ± 0.73	0.850
TG (mmol/L)	1.17 ± 0.23	1.17 ± 0.22	0.865
LDLC (mmol/L)	2.94 ± 0.51	2.88 ± 0.52	0.210
HDLC (mmol/L)	1.07 ± 0.23	1.09 ± 0.25	0.313
CRP (pg/mL)	1479.88 ± 140.21	1484.79 ± 136.43	0.721
IL-6 (pg/mL)	19.31 ± 4.17	19.57 ± 3.78	0.511
TNF-*α* (pg/mL)	79.09 ± 8.10	79.04 ± 8.20	0.950
IL-11 (pg/mL)	87.72 ± 13.64	87.24 ± 13.61	0.728

*P* comparison between THE RT group and control group. Continuous data presented nonnormal distribution (age, NIHSS, and infarct volume) were expressed by median (range) and analyzed by Mann–Whitney *U* test. Continuous data presented normal distribution (BMI, FPG, TC, TG, LDLC, HDLC, CRP, IL-6, TNF-*α*, and IL-11) were expressed by mean ± SD and analyzed by Student's *t* test. Chi-square test was used for rates (sex, current smoker, complication, and infarct location).

**Table 2:** Correlation analysis among IL-11, NIHSS, and mRS.

	IL-11	NIHSS	mRS
IL-11			
Spearman's correlation	1	-0.118	-0.107
*P*		0.018	0.032
NIHSS			
Spearman's correlation	-0.118	1	0.267
*P*	0.018		<0.001
mRS			
Spearman's correlation	-0.107	0.267	1
*P*	0.032	<0.001	

**Table 3:** Serum IL-11 levels and clinical data in ischemic stroke patients with different prognoses.

Variable	mRS score ≤ 2 group (*n* = 256)	mRS score ≥ 3 group (*n* = 148)	*P*
Age, y	58 (44~74)	59 (41~79)	0.567
Sex, female (%)	133 (51.95)	66 (44.59)	0.396
BMI	25.75 ± 2.12	25.38 ± 2.24	0.098
Current smoker, *n* (%)	97 (37.89)	64 (43.24)	0.565
NIHSS	5 (1~22)	14 (1~20)	<0.001
Hypertension, *n* (%)	178 (69.53)	109 (73.65)	0.637
Hyperlipidemia, *n* (%)	45 (15.78)	21 (14.19)	0.563
Diabetes, *n* (%)	57 (22.27)	32 (21.62)	0.999
Infarct volume (cm^3^)	0.80 ± 0.30	2.85 ± 1.31	<0.001
Infarct location			
Left, *n* (%)	137 (53.12)	85 (57.43)	0.776
Right, *n* (%)	79 (30.86)	59 (39.86)	0.237
Both sides, *n* (%)	27 (10.55)	17 (11.49)	0.999
FPG (mmol/L)	6.83 ± 0.59	6.82 ± 0.58	0.961
TC (mmol/L)	4.33 ± 0.72	4.29 ± 0.75	0.600
TG (mmol/L)	1.14 ± 0.21	1.22 ± 0.23	0.001
LDLC (mmol/L)	2.92 ± 0.50	2.88 ± 0.52	0.467
HDLC (mmol/L)	1.05 ± 0.17	1.12 ± 0.31	0.005
CRP (pg/mL)	1476.25 ± 136.38	1493.00 ± 140.95	0.241
IL-6 (pg/mL)	17.43 ± 2.83	22.91 ± 3.17	<0.001
TNF-*α* (pg/mL)	78.26 ± 8.05	80.43 ± 8.13	0.010
IL-11 (pg/mL)	95.60 ± 6.26	73.41 ± 11.22	<0.001

*P* comparison between the mRS score ≤ 2 group and mRS score ≥ 3 group. Continuous data presented nonnormal distribution (age and NIHSS) were expressed by median (range) and analyzed by Mann–Whitney *U* test. Continuous data presented normal distribution (BMI, FPG, TC, TG, LDLC, HDLC, CRP, IL-6, TNF-*α*, IL-11, and infarct volume) were expressed by mean ± SD and analyzed by Student's *t* test. Chi-square test was used for rates (sex, current smoker, complication, and infarct location).

**Table 4:** Risk factors of ischemic stroke patients with bad prognosis by logistic regression analysis.

Variables	Wald	Odds ratio	95% CI	*P*
Age	0.173	1.007	03947~1.041	0.678
BMI	2.902	0.888	0.775~1.018	0.088
FPG	0.235	1.131	0.687~1.864	0.628
NIHSS	15.728	1.664	1.294~2.139	<0.001
Infarct volume	12.203	43.853	5.257~365.819	<0.001
TC	1.913	0.748	0496~1.129	0.167
TG	4.264	4.096	1.074~15.615	0.039
HDLC	4.148	3.591	1.049~12.290	0.042
LDLC	0.798	1.309	0.725~2.364	0.372
CRP	1.027	1.001	0.999~1.003	0.311
IL-6	89.145	1.891	1.657~2.159	<0.001
TNF-*α*	0.903	1.017	0.982~1.054	0.342
IL-11	9.052	0.526	0.346~0.799	0.003

## Data Availability

The datasets used and analyzed during the current study are available from the corresponding authors on reasonable request.
